# Inhibition of pathogenic and spoilage bacteria by a novel biofilm-forming *Lactobacillus* isolate: a potential host for the expression of heterologous proteins

**DOI:** 10.1186/s12934-015-0283-8

**Published:** 2015-07-07

**Authors:** Tannaz Jalilsood, Ali Baradaran, Adelene Ai-Lian Song, Hooi Ling Foo, Shuhaimi Mustafa, Wan Zuhainis Saad, Khatijah Yusoff, Raha Abdul Rahim

**Affiliations:** Department of Cell and Molecular Biology, Faculty of Biotechnology and Biomolecular Sciences, Universiti Putra Malaysia, UPM, 43400 Serdang, Selangor Malaysia; Department of Bioprocess Technology, Faculty of Biotechnology and Biomolecular Sciences, Universiti Putra Malaysia, UPM, 43400 Serdang, Selangor Malaysia; Institute of Bioscience, Universiti Putra Malaysia, UPM, 43400 Serdang, Selangor Malaysia; Department of Microbiology, Faculty of Biotechnology and Biomolecular Sciences, Universiti Putra Malaysia, UPM, 43400 Serdang, Selangor Malaysia; Halal Products Research Institute, Universiti Putra Malaysia, UPM, 43400 Serdang, Selangor Malaysia; Institute of Tropical Forestry and Forest Products, Universiti Putra Malaysia, UPM, 43400 Serdang, Selangor Malaysia

**Keywords:** Biofilm, *Lactobacillus plantarum*, Food-borne pathogens, Microbial cell factory, *gfp*

## Abstract

**Background:**

Bacterial biofilms are a preferred mode of growth for many types of microorganisms in their natural environments. The ability of pathogens to integrate within a biofilm is pivotal to their survival. The possibility of biofilm formation in *Lactobacillus* communities is also important in various industrial and medical settings. Lactobacilli can eliminate the colonization of different pathogenic microorganisms. Alternatively, new opportunities are now arising with the rapidly expanding potential of lactic acid bacteria biofilms as bio-control agents against food-borne pathogens.

**Results:**

A new isolate *Lactobacillus plantarum* PA21 could form a strong biofilm in pure culture and in combination with several pathogenic and food-spoilage bacteria such as *Salmonella enterica*, *Bacillus cereus, Pseudomonas fluorescens,* and *Aeromonas hydrophila*. Exposure to *Lb. plantarum* PA21 significantly reduced the number of *P. fluorescens,A. hydrophila* and *B. cereus* cells in the biofilm over 2-, 4- and 6-day time periods. However, despite the reduction in *S. enterica* cells, this pathogen showed greater resistance in the presence of PA21 developed biofilm, either in the planktonic or biofilm phase. *Lb. plantarum* PA21 was also found to be able to constitutively express GFP when transformed with the expression vector pMG36e which harbors the *gfp* gene as a reporter demonstrating that the newly isolated strain can be used as host for genetic engineering.

**Conclusion:**

In this study, we evaluate the ability of a new *Lactobacillus* isolate to form strong biofilm, which would provide the inhibitory effect against several spoilage and pathogenic bacteria. This new isolate has the potential to serve as a safe and effective cell factory for recombinant proteins.

## Background

Bacterial biofilms are a natural complex of microorganisms embedded in a protective slimy matrix composed of various types of polysaccharides, proteins, nucleic acids and lipids [1]. The ability to form a biofilm is an important property for both pathogenic bacteria and bacteria used in diverse processes, such as fermentation and/or the preservation of food and feed. Biofilms are resistant to antimicrobial agents and present major challenges in the application of disinfectant treatments [2]. The food industry faces serious challenges due to equipment impairment caused by metal corrosion in pipelines resulting from chemical and biological reactions by resident biofilms [3–5].

The adhesion capacity of food and water-borne pathogens, such as *Salmonella* spp., *Bacillus cereus*, *Pseudomonas fluorescens* and *Aeromonas hydrophila,* which develop biofilms in food-processing plants, lead to the transmission of diseases and decreased product shelf-life [6–10]. Some lactic acid bacteria (LAB) were discovered to have positive properties that could be used to control various types of pathogens and spoilage microorganisms [11, 12]. Lactic acid bacteria are well known as beneficial bacteria and include probiotic bacteria that have positive effects on the prevention of gastrointestinal related diseases improving digestion in lactose intolerants by alleviating it [13], preventing intestinal tract infections [14], reducing inflammatory or allergic reactions [15, 16], and easing the absorption of nutrients [17, 18]. Due to their health-promoting properties, LAB, particularly lactobacilli, are valued as candidates for cancer therapy, vaccine delivery, and immune-modulators [19].

The main feature of LAB, notably *Lactobacillus*, is their ability to ferment sugars leading to many organic acids production such as lactic, acetic and propionic acids as end products which provide an acidic environment unfavorable for the growth of many pathogenic and spoilage microorganisms [20]. LAB are well adapted to live in low pH and high lactic acid environments [21, 22], and are therefore key players in fermented food ecosystems [23, 24]. The use of lactic acid bacteria and their metabolites is the most common and popular in methods of natural protection. In addition, biofilms are yet another protective agent formed by lactic acid bacteria. Current biofilm preventive strategies by *Lactobacillus* against pathogenic bacteria are essentially aimed with production of antimicrobial metabolites or inhibitory extracellular polymeric substance (EPS) surrounding the pathogenic bacteria. However, recent studies suggested that competition for adhesion sites and nutrients could also interfere with biofilm formation in pathogenic organisms, modulating *Lactobacillus*-pathogen interfaces [25]. To date, few studies have addressed this issues in multispecies biofilm context; new information on *Lactobacillus* interactions with mixed biofilm communities is therefore needed.

Previously, it has been shown that biofilm formation and dispersal are regulated by several key regulatory proteins. These core proteins involved in the synthesis of adhesions and biofilm matrix components are evidently known, providing a tool for biofilm formation control [1]. Engineering of even more efficient biofilm producers may be achieved by manipulating metabolic pathways via overexpression or down-regulation/knock-out of specific target proteins, which can mediate cell-to-cell interconnections or promote early biofilm formation and thereby bacterial survival.

As such, in the present study, apart from evaluating the effectiveness of the new *Lactobacillus* isolate with adhesive properties to inhibit several pathogenic and food-spoilage bacteria, we also verified the ability of this strain to function as a host for future genetic engineering work. This is anticipated to improve biofilm production in this strain and provide insights regarding different aspects of the adhesion process.

## Results

### Identification of LAB species derived from *Pandanus* leaves

**Figure 1:**
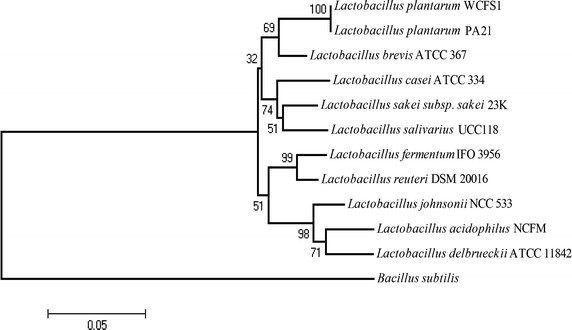
A phylogenetic tree was constructed with the MEGA version 4.1 program using 16S rDNA gene sequences. Data for 16S rDNA phylogenetic analysis were obtained from the Genbank nucleotide sequence database for the following strains: *Lactobacillus sakei subsp. sakei* 23k (GenBank accession no. NC_007576), *Lactobacillus casei* ATCC 334 (GenBank accession no. CP000423), *Lactobacillus salivarius* UCC 118 (GenBank accession no. NC_007929), *Lactobacillus acidophilus* NCFM (GenBank accession no. NC_006814), *Lactobacillus plantarum* WCSF1 (GenBank accession no. AL935263), *Lactobacillus reuteri* JCM 1112 (GenBank accession no. NC_009513), *Lactobacillus brevis* ATCC 367 (GenBank accession no. NC_008497), *Lactobacillus fermentum* IFO 3956 (GenBank accession no. NC_010610), *Lactobacillus reuteri* DSM 20016 (GenBank accession no. NC_009513), *Lactobacillus johnsonii* NCC 533 (GenBank accession no. NC_005362), and *Lactobacillus delbruekii* ATCC 11842 (GenBank accession no. NC_008054). *Bacillus subtilis* NCDO 1769 (GenBank accession no. NC_000964) was used as an out-group organism. The *bar* indicates the number of nucleotide substitutions per site. The robustness of the NJ tree was tested by bootstrapping with 1,000 replicates of data, and percentages are reported at the nodes (only values above 50% are reported).

The combination of biochemical and Gram stain results led to the identification of several putative LAB. The rapid crystal violet microtiter plate adherence test showed that one of the isolates was more efficient in its attachment to the well surface of microtiter plates compared with the *Lactobacillus. plantarum* ATCC 14917 control strain. Based on the 16S rDNA gene sequences, the best biofilm producer was identified as *Lb. plantarum* and designated as *Lb. plantarum* PA21. The comparison of the 16s rDNA gene sequences differentiated *Lb. plantarum* PA21 from other major bacteria in the same genus. The phylogenetic tree based on 16s rDNA was constructed; relationships among top hits after BLASTN similarity searches were identified. The *Lb. plantarum* PA21 was found to be closely related to *Lb. plantarum* WCFS1 (Figure 1). The 16S rDNA sequence in this study was submitted to GenBank under the accession number JX244277.

### Analysis of LAB biofilm formation

**Figure 2:**
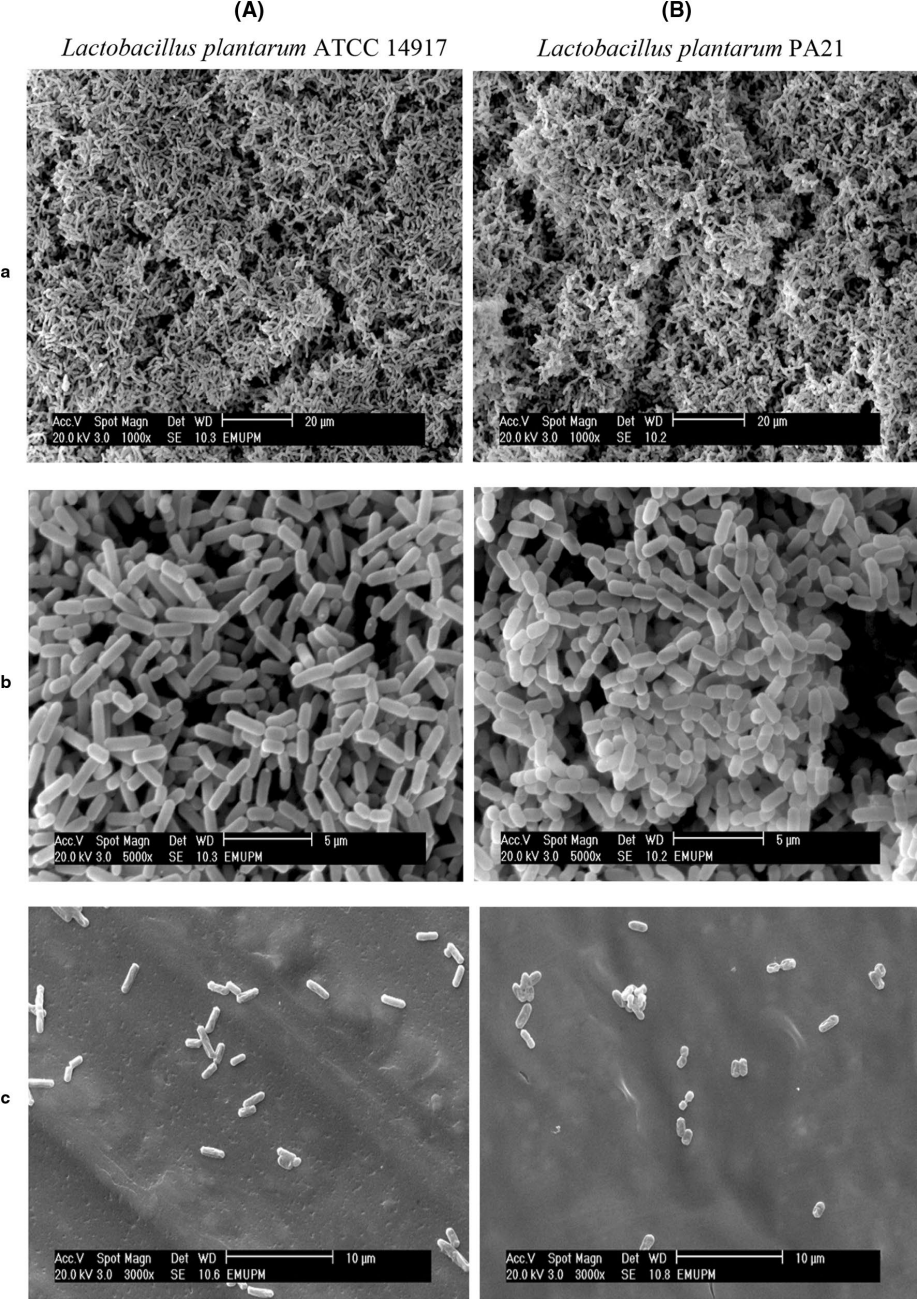
Comparative analysis of *Lactobacillus plantarum* PA21 in biofilm and planktonic culture. SEM analysis of *a* biofilm covered-surfaces, *b* biofilm cells, and *c* planktonic cells of **A** *Lactobacillus plantarum* ATCC 14917, **B** *Lactobacillus plantarum* PA21 in MRS broth after 24 h at 35°. *Lactobacillus plantarum* ATCC 14917 was used as positive control.

**Figure 3:**
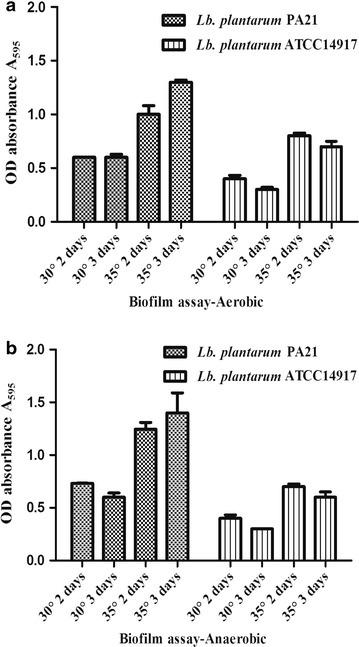
Biofilm formation on microtitre plates. Biofilm formation by two *Lactobacillus* strains on polystyrene microtitre plates following growth at 2 and 3 days at 30° and 35° in aerobic (**a**) and anaerobic (**b**) conditions in MRS broth: biofilms were stained with crystal violet, de-stained using 95% alcohol and the optical density at 595 nm of the alcoholic crystal violet solutions determined (OD optical density). Assays were performed three times for all 2 strains. *Bars* represent average values and standard errors of three observations.

SEM allowed the visualization of biofilm surface structures with a three-dimensional appearance at very different resolutions (Figure 2) and has been reported as an indirect method to estimate bacteria in situ [26, 27]. The ability of the *Lactobacillus* isolate to form the biofilm was also determined at two different temperatures and classified based on temperature and aeration (Figure 3). The mean of the resultant optical density was significant after 3 days at 35°C under both aerobic (Figure 3a) and anaerobic (Figure 3b) conditions. A microtiter plate adherence test and the imaged biofilms showed that the newly isolated *Lactobacillus* could form a strong biofilm.

### Antibiotic susceptibility test

The resistance of the new *Lactobacillus* isolate was scored via agar disc diffusion on MRS agar medium. The strain displayed phenotypic resistance, which is a general feature of this species (Table 1). *Lb. plantarum* PA21 was susceptible to β-lactam antibiotics, which include penicillin G. The isolate was also susceptible to erythromycin, chloramphenicol, bacitracin, clindamycin and tetracycline. In addition, it was resistant to quinolones (nalidixic acid), glycopeptides (vancomycin) and aminoglycosides, which include kanamycin and streptomycin.

**Table 1:** Antibiotic susceptibility of *Lb. plantarum* PA21 analysed using agar-disc diffusion method

Antibiotic	Concentration	Target	Diameter of inhibition zone of strains (mm) *Lb. plantarum* PA21/*Lb. plantarum* ATCC 14917	
Aminoglycoside		Ribosome		
Kanamycin	30 μg		–	–
Streptomycin	10 μg		–	–
Penicillin/β-lactam		Cell wall		
Penicillin G	10 units		20	15
Macrolide		Ribosome		
Erythromycin	15 μg		18	22
Polymyxins		Cell membrane		
Bacitracin	10 units		14	14
Phenicole		Ribosome		
Chloramphenicol	30 μg		17	20
Quinolone		DNA topoisomerase		
Nalidixic acid	30 μg		–	–
Tetracycline		Ribosome		
Tetracycline	30 μg		15	15
Glycopeptides		Cell wall		
Vancomycin	30 μg		–	–
Lincosamides		Ribosome		
Clindamycin	10 μg		11	18

Susceptibilities were evaluated by measuring (in mm) zones of growth inhibition in standard disc diffusion assay. *Lb. plantarum* PA21 is resistant to kanamycin, streptomycin, nalidixic acid and vancomycin.

### Antipathogenic activity of LAB biofilms

The viable counts of *S. enterica, B. cereus, P. fluorescens* and *A. hydrophila* cells that survived in biofilm (Table 2) and planktonic forms (Table 3) in the presence of *Lb. plantarum* PA21 were observed after 2, 4 and 6 days without replenishing nutrient. The effects of three main factors, P = Pathogen, L = LAB, and T = Time, were analyzed in a factorial experiment with two or more levels for each factor: *S. enterica, B. cereus, P. fluorescens* and *A. hydrophila* for pathogens; *Lb. plantarum* PA21 for LAB; and 2, 4 and 6 days for time were selected as different levels to measure the response in various combinations of factors and levels (PL, PT, LT). The results indicated the factors that have the largest effect on the response and possible interactions between factors. Based on the relationship between the pathogens and time levels (PT), the number of biofilm and planktonic cells were lowest for *B. cereus* during the first 2 days, while these parameters were highest for *S. enterica* during the same period. The mean results identified a linear decrease in both the biofilm and planktonic forms for all pathogens in the presence of *Lb. plantarum* PA21. The PL, LT and PLT combinations indicate that the overall means of the growth rate were highest in the positive control; this finding confirmed the significant effect of *Lb. plantarum* PA21 in controlling pathogens in biofilm and planktonic forms. In the presence of PA21, the planktonic and biofilm cells of *S. enterica* persisted even after 6 days, albeit at reduced levels. The interaction between the three variables (PLT) also indicated that both *S. enterica* biofilm and planktonic cells were reduced after 6 days by 2.4 log in the adherent and 3.86 log in the planktonic culture, while the mean for *Salmonella* remained higher than the mean for all other pathogens present at the same time compared to the control (p ≤ 0.01). The numbers of *P. fluorescens,A. hydrophila* and *B. cereus* cells in the biofilm were reduced on the second day of incubation by 1.7 log, 2.2 log and 5.9 log (p ≤ 0.01), respectively, and not detectable at the end of the experiments.

**Table 2:** Effects of *Lactobacillus plantarum* PA21 biofilm on the planktonic cell viability of food spoilage and pathogenic bacteria at 2 days intervals (means log_10_ CFU/ml ± SD)

Strains	Viable counts of planktonic cells (CFU/ml)		
	2 days	4 days	6 days
*S. enterica*	5.75 ± 0.01	6.59 ± 0.05	4.87 ± 0.01
*P. fluorescens* ATCC 13525	2	nd	nd
*A. hydrophila* ATCC 7965	4.2 ± 0.02	nd	nd
*B. cereus*	5.53 ± 0.01	nd	nd
*S. enterica* ^a^	8.95 ± 0.005	8.39 ± 0.04	8.73 ± 0.01
*P. fluorescens* ATCC 13525^a^	8.94 ± 0.03	8.58 ± 0.01	7.88 ± 0.03
*A. hydrophila* ATCC 7965^a^	8.01 ± 0.02	8.88 ± 0.03	8.57 ± 0.04
*B. cereu*s^a^	7.80 ± 0.01	7.02 ± 0.02	7.63 ± 0.01

At day 0, all pathogens were added to yield a final bacterial count of approximately 6-log_10_ CFU/ml.

*nd* Not detected.

^a^strains used as a positive control.

**Table 3:** Preventive effects of *Lactobacillus plantarum* PA21 biofilm on the attachment of food spoilage and pathogenic bacteria at 2 days intervals (means log_10_ CFU/ml ± SD)

Strains	Viable counts of biofilm cells (CFU/cm^2^)		
	2 days	4 days	6 days
*S. enterica*	5.51 ± 0.02	5.19 ± 0.005	4.58 ± 0.03
*P. fluorescens* ATCC 13525	4.11 ± 0.01	2. 88 ± 0.03	nd
*A. hydrophila* ATCC 7965	4.59 ± 0.05	nd	nd
*B. cereus*	nd	nd	nd
*S. enterica* ^a^	7.46 ± 0.01	7.22 ± 0.03	6.98 ± 0.008
*P. fluorescens* ATCC 13525^a^	5.82 ± 0.03	6.34 ± 0.006	5.94 ± 0.03
*A. hydrophila* ATCC 7965^a^	6.84	7.35 ± 0.03	6.74 ± 0.01
*B. cereu*s^a^	5.90 ± 0.02	6.06 ± 0.07	6.14 ± 0.08

At day 0, all pathogens were added to yield a final bacterial count of approximately 6-log_10_ CFU/ml.

*nd * Not detected.

^a^strains used as a positive control.

To better characterize the inhibitory effect of PA21 biofilm, the viable counts of pathogen cells were also measured in the absence of PA21 biofilm structure, using only PA21 planktonic cells (Table 4). While PA21biofilm was not structured, it has become apparent that viable cell counts of *P. fluorescens,A. hydrophila* and *S. enterica* cells were also reduced in 2 days of incubation in mixed bacterial cultures by 3.4, 3.0 and 1.9 log (p ≤ 0.01) respectively; however, pathogen cells showed considerably lower numbers when co-cultured with formed PA21biofilm cells in the same time period except for *B. cereus*. Together, these results determined how the structured environment of PA21 biofilm would alter inhibitory effect on the growth of pathogens cultures.

**Table 4:** Effects of *Lactobacillus plantarum* PA21 in the absence of PA21 biofilm structure on the cell viability of food spoilage and pathogens bacteria at 2 days intervals (means log_10_ CFU/ml)

Strains	Viable counts of planktonic cells (CFU/ml)		
	2 days	4 days	6 days
*S. enterica*	7.10 ± 0.01	6.34 ± 0.01	4.6 ± 0.05
*P. fluorescens* ATCC 13525	5.48 ± 0.03	2.28 ± 0.03	nd
*A. hydrophila* ATCC 7965	5.94 ± 0.02	nd	nd
*B. cereus*	4.60 ± 0.01	nd	nd
*S. enterica* ^a^	8.95 ± 0.005	8.39 ± 0.04	8.73 ± 0.01
*P. fluorescens* ATCC 13525^a^	8.94 ± 0.03	8.58 ± 0.01	7.88 ± 0.03
*A. hydrophila* ATCC 7965^a^	8.01 ± 0.02	8.88 ± 0.03	8.57 ± 0.04
*B. cereu*s^a^	7.80 ± 0.01	7.02 ± 0.02	7.63 ± 0.01

At day 0, all pathogens were added to yield a final bacterial count of approximately 6-log_10_ CFU/ml.

*nd* Not detected.

^a^strains used as a positive control.

### LAB biofilm maturation and dispersal

**Figure 4:**
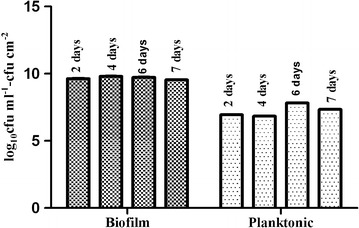
Biofilm maturation and planktonic cell growth of *Lactobacillus plantarum* PA21. Cell viabilities of biofilm and planktonic *Lactobacillus plantarum* PA21 were measured over 7 days of biofilm development.

When considering the competitive-inhibition of *Lb. plantarum* PA21 and different pathogens, the cells density of *Lb. plantarum* PA21 needs to be determined in the period of biofilm maturation as well as by co-culture experiments. The direct comparison of the adhesion ability of tested *Lactobacillus* was possible when using quantitative analyses to assess the relative biofilm densities. Prior to adding pathogens, the viable cell counts (log CFU) positively correlated with the days of incubation from day 1 to 4, which indicated that a *Lb. plantarum* PA21 biofilm formed. The *Lb. plantarum* PA21 cell counts analyses (CFU/cm^2^) revealed the highest biofilm cell density after 4 days of culture (Figure 4). A slight decrease in the viable cell count was observed in 6-day-old biofilm cells. On the contrary, the lowest and the largest numbers of planktonic cells were observed after 4 and 6 days, respectively. As the biofilm ages, the attached bacteria must be able to detach and disperse from the biofilm in order to survive and colonize new niches.

**Figure 5:**
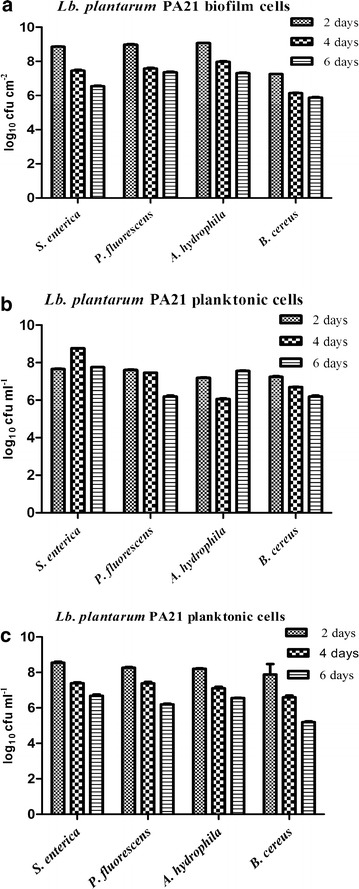
Number of viable *Lactobacillus plantarum* PA21 cells recovered from biofilm and planktonic cultures after contact with pathogens and food spoilage bacteria. Viable counts of *Lb. plantarum* PA21 biofilm cells (**a**), biofilm shed planktonic cells (**b**) and wild-type planktonic cells (**c**) were measured in co-cultures with *Salmonella enterica, Pseudomonas fluorescens* ATCC 13525, *Aeromonas hydrophila* ATCC 7965 and *Bacillus cereus.* *Error bars* indicate standard deviations of three independent experiments.

The recovery (CFU) of *Lactobacillus* biofilm cells in combination with each of the above-mentioned pathogens was also calculated. Because the *Lb. plantarum* PA21 biofilm contributed to reducing the growth of pathogens, we sought to determine the major impact on the mean values of *Lb. plantarum* PA21 biofilm and planktonic cells by these pathogens compared to the PA21 negative control. After 2 days of incubation, all of the pathogens tested appeared to exert almost no effect on the biofilm log reduction of *Lb. plantarum* PA21, except for *B. cereus* (Figure 5a). The number of cells in the *Lactobacillus* biofilm, which was initially 9 × 10^7^ CFU/cm^2^, was reduced by 1.77 log in the presence of *B. cereus*. The number of biofilm cells gradually decreased over time, and the maximum reduction of 3.2 log was registered in the presence of *B. cereus* for 6-day-old biofilm. Within this interval, *P. fluorescens* exerted the smallest effect on *Lactobacillus* biofilm cells with only a 1.7 log reduction.

The differences in the response to various pathogens in biofilm-derived planktonic cells are also shown in Figure 5b. For PA21planktonic cells that were shed from biofilm, a similar trend was found in the presence of *P. fluorescens* and *B. cereus*, i.e., decreasing mean values at the end of the experiments compared to the first 2 days by 1.4 and 1.0 log, respectively. Interestingly, the trend for the bacterial densities (CFU/ml) in planktonic form was not consistent in the presence of biofilm cultures of *S. enterica* and *A. hydrophila.* The highest and lowest cell counts were observed after 4 days of incubation for *S. enterica,* and *A. hydrophila*, respectively, although the mean survival values of planktonic cells in the first 2 days and at the end of experiments were almost the same.

Moreover, the viability of wild-type PA21 planktonic cells that had not adhered to a surface was also compared with planktonic cells yielded from biofilm in mixed culture with spoilage and pathogenic bacteria. The obtained results showed greater numbers at day 2, while they were gradually decreased over 6 days starvation. The lowest number of planktonic cells was observed at day 6 in the presence of *B. cereus.* Comparison of viable cell counts results suggested that the survival values of planktonic cells were significantly lower in the absence of structured PA21 biofilm (Figure 5c).

### Effect of antimicrobial metabolites

Lactobacilli reveal different antimicrobial mechanisms which can be shown through in vitro assays. The antimicrobial activity in liquid media is favored by rapidly diffusing antimicrobial compounds including organic acids and bacteriocins [28]. In the present study no inhibitory effect of bacteriocin was observed against the pathogens and spoilage bacteria previously listed (data not shown).

**Figure 6:**
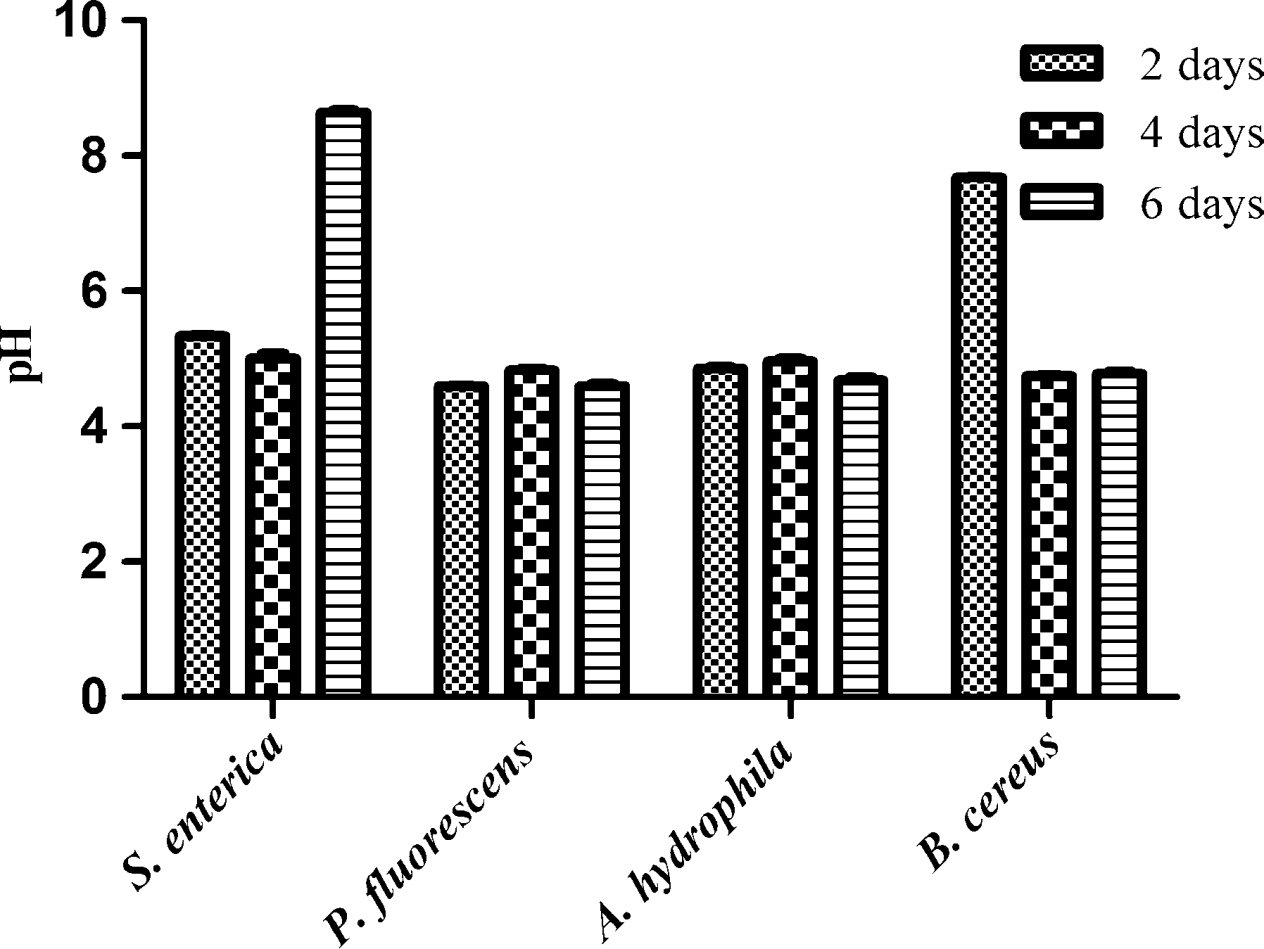
Measure pH changes. pH values were measured during 6 days cultivation of *Salmonella enterica, Pseudomonas fluorescens* ATCC 13525, *Aeromonas hydrophila* ATCC 7965 and *Bacillus cereus* with *Lb. plantarum* PA21 biofilm.

In order to illustrate the possible influence of organic acid, a pH measurement was required. The new isolate produced a low content of lactic acid of 0.5 g/L in overnight culture, while acetate production was approximately 0.28 g/L. During the production of organic acids, the pH in the fermentation broth gradually decreased to 3.51 ± 0.02 in the first 2 days of biofilm development and it was constant during 7 days biofilm maturation. Interestingly, pH values lower than those in the control were found in the first 2 days of exposing *Lb. plantarum* PA21 biofilm to all of the above-mentioned pathogens except *B. cereus*. In addition, a pH level above 8 was observed after 6 days of incubation in the presence of *S. enterica* (Figure 6). The same pH patterns were observed within all above mentioned- pathogens in the absence of PA21biofilm (data not shown).

### Verification of *Lb. plantarum* transformants

The total DNA isolation of *Lb. plantarum* PA21 compared to the control strain *Lb. plantarum ATCC* 14917 showed that the strain was devoid of the low molecular weight plasmid (results not shown). Based on the plasmid bands extracted from *Lb. plantarum* PA21 transformants, this new host can carry and replicate pMG36e containing the GFP insert without any indication of possible incompatibility. GFP gene could be retrieved from the digested recombinant plasmid pMG36e-GFP.

To calculate the generation time, the growth curves were obtained and viability plating was performed, and revealed a 48-min doubling time for *Lb. plantarum* PA21 carrying pMG36e-GFP. PA21 transformants carrying Em^r^ on plasmid pMG36e were grown for 100 generations without antibiotic selection. After 100 generations without selective pressure, the number of viable cells growing on medium containing erythromycin suggested that the *gfp*-marked plasmid was 100% stable in *Lb. plantarum* PA21.

### Expression of heterologous protein

**Figure 7:**
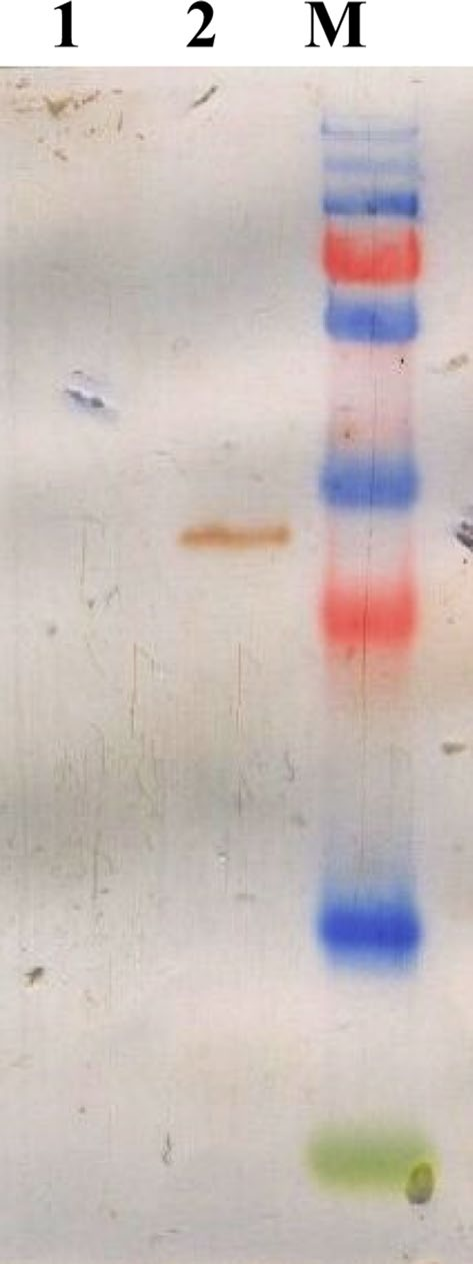
Western blot analysis. Lanes M, PageRuler™ Plus Prestained Protein Ladder; Lanes 1, Empty *Lactobacillus plantarum* PA21 (Negative control); Lane 2, pMG36e-GFP clone.

The GFP expression plasmid was constructed by placing the *gfp* gene downstream of the constitutive promoter P32 and propagating the resultant plasmid within *Lb. plantarum* PA21. The plasmid was extracted, purified and digested to confirm the presence of the insert (GFP) by comparing the size differences of the linearized plasmid. A comparison of the PCR method and double digestion of the recombinant plasmid indicated the presence of the inserted DNA fragment. GFP expression was confirmed by the presence of a 27-kDa protein band on the blotting membrane following SDS-PAGE and western blot (Figure 7).

## Discussion

This study aimed to select an isolate of *Lactobacillus* spp. from the leaves of *Pandanus amaryllifolius* with the potential for biofilm development to inhibit various types of food-borne spoilage and pathogenic bacteria. The applicability and usefulness of the newly isolated strain, denoted as *Lb. plantarum* PA21, were extended via its capacity to express heterologous protein. The imaged biofilms and cell count results showed differences during the biofilm maturation periods. The ability of bacteria to adhere to the abiotic surface in plastic microtiter plates was measured using a conventional biofilm assay. The method offers some advantages compared to the study of biofilm formation in flow cells, which is an alternative widely used method. Watnick and Kotler [29] showed that the microtiter plate assay can be utilized to distinguish true biofilm formation similar to the biofilm grown in flow cells. This method appeared attractive for obtaining quantitative results based on CFU and optical density.

Previous study has shown that the ability of bacteria to integrate within a biofilm is basic to their survival. Importantly, the temperature, availability of nutrients, pH level, contact time of the bacteria with the surface, growth stage, and surface hydrophobicity can affect the development of biofilm [25]. Biofilm formation by *Lb. plantarum* PA21 was measured at 30°C and subsequently enhanced by increasing the temperature to 35°C. Higher temperatures have been suggested to increase the initial adherence of LAB cells by promoting the generation and secretion of extracellular polymeric substances, which increases the biofilm density [30]. The plating of *Lb. plantarum* PA21 biofilm cultures demonstrated no significant change in the adherent population after 4 days of incubation, suggesting that the new isolate could form a mature biofilm after 4 days.

In the natural environment and in the presence of other bacteria, 95–99% of microorganisms show biofilm-forming capabilities. Numerous studies have reported that pathogens may be protected when living in association with other strains in a mixed biofilm [31]. In some food related environments, when a planktonically grown pathogen lands on a surface, it encounters the interface of a resident biofilm rather than a sterile material. LAB were reported to be good candidates to settle protective positive biofilms on food processing environment, a key role in controlling colonization by competitive interactions with food pathogens [11, 32]. The presence of *Listeria monocytogenes* in the biofilm on the surfaces of food-processing plants was controlled by bacteriocin-producing *Lactococcus lactis*, suggesting that LAB can be used as a “house microflora” to suppress the establishment of enteropathogens in a food-processing environment [33]. Based on known facts about LAB biofilm and its behavior, the concept of “protective cultures” is a broad one and is not strictly related to the production of bacteriocins and organic acids, whose antimicrobial action is well known. Competition of protective cultures with potential pathogens is another major contributor to eliminate the primary localization of undesired organisms on the surface.

To assess the inhibition spectrum of strain PA21, a biofilm study was carried out to determine the ability of PA21 to form a biofilm in response to various types of pathogens. For this purpose, a 6- to 7-day period allowed the new isolate to grow and mature into a biofilm [11]. The cell count results over 7 days of biofilm maturation demonstrated that the adhesion capability of *Lb. plantarum* PA21 was high, which can be utilized to protect surfaces during maturation. Viability of *Lactobacillus* isolate in a dual-species culture with pathogen species revealed strong biofilm during the first 2 days. Furthermore, there appeared to be a shift in the PA21 biofilm pattern from strong to moderate after 2 days and this may probably be due to a combination of factors, including inhibition of the growth stage, nutrient depletion and activity of either organism on the other.

Along with PA21 adherence pattern, the positive effect of this strain was also important for control or inhibition biofilm formation by pathogenic organisms. When the pathogens were challenged with PA21 for adhered and planktonic cells, the decrease in viable counts was strongly correlated with the presence or absence of PA21 biofilm. Due to *Lb. plantarum* PA21’s remarkable ability to inhibit the growth of pathogens, the viable counts of *A. hydrophila*, *P. fluorescens* and *B. cereus* biofilm cells were significantly reduced in the first 2 days, and no countable cells were detected at the end of the experiments, although *S. enterica* was able to survive in the presence of *Lb. plantarum* PA21 during 6 days compared to *A. hydrophila* and *P. fluorescens.* These differences were possibly attributed to *S. enterica* either having better carbon and nitrogen metabolism under nutrient-limited condition, or activating tolerance response to acid stress in order to survive multiple detrimental environmental factors [34]. Moreover, in the presence of *Salmonella*, the endpoint of the 6 days incubation was the production of an alkaline pH. As previously observed, *Salmonella* rapidly metabolized glucose; as glucose depletes, the peptones (amino acids) are aerobically utilized as an energy source. Utilization of peptones causes the release of ammonia (NH_3_) resulting in the alkaline pH [35]. *Salmonella* spp. may also increase their internal pH when they are exposed to a lethal pH challenge. Álvarez-Ordóñez et al. [36] demonstrated that *Salmonella* spp. can cope with the acid challenges encountered in various ecological niches, such as the environment in food processing plants and the gastrointestinal tract, via the log-phase and stationary-phase adaptive acid tolerance response (ATR) when organic acid is used. A potential ATR has also been proven in *Aeromonas* subjected to a low pH similar to that exhibited in some important enteric pathogens, including *Salmonella enterica* serovar *Typhimurium* and *E. coli*. San Jose et al. [37] reported that in addition to utilizable C and N sources, *Pseudomonas* can use lactic acid as a source of carbon and energy during biofilm formation while interacting with *Lactococcus lactis* subsp*. cremoris.* They can also obtain additional nutrients from the autolysis of lactococci [38, 39]. However, acid tolerance may be strain dependent and this may explain the differences between the planktonic and biofilm populations of *S. enterica, A. hydrophila* and *P. fluorescens* in the present of PA21 during 6 days incubation.

*B. cereus* was also found capable of producing alkaline pH for the first 2 days. *B. cereus* was grown in nutrient broth composed of a simple peptone and a beef extract. Peptone contributes organic nitrogen in the form of amino acids; alkaline pH was likely due to the formation of ammonium from ammonia resulting in an elevation of the pH from acidic pH, and/or availability of nitrogen [40]. *B. cereus* was only detected in the planktonic form on day 2, after which it could no longer be cultured. Interestingly, at the same time, the lowest number of PA21 biofilm cells was also detected in co-culture with *B. cereus. B. cereus* can use enzymes, such as amylase and protease, as a defense to break down existing biofilm instead of only killing planktonic organisms [41]. Notably, most bacillus proteases are active in a neutral or alkaline pH [42].

To determine the feasibility of using *Lactobacillus* as recombinant host for biological control strategies against different food-borne pathogens, it must be able to express genes of interest under inducible or constitutive expression systems [43]. It has been brought to light that quorum sensing (QS) is a chemical signaling systems that control biofilm formation in bacteria [44]. Quorum sensing is a cell density-dependent signaling system that coordinates many bacterial activities through small signal molecules known as autoinducers (AI). Many of the QS regulated microbial activities are involved in food spoilage and survival of pathogens within the food matrix. Proteomic analysis revealed that *Lactobacillusacidophilus* downregulates biofilm formation by reducing the AI-2 activity of *E. coli* O157:H7 [45]. Interventions targeting bacterial QS in food are currently largely unexplored. Biocontrol strategies that exploit bacterial QS provide an opportunity to alter microbial activity such that survival of targeted microorganism is unlikely. This property can be enhanced with the help of genetic engineering as several key proteins have been shown to block QS by degrading the signal, signal analogues and signal antagonist [46].

To accomplish this goal, verification of PA21 as an expression host was performed. Plasmid pMG36e, carrying the *gfp* gene under the control of the constitutive p23 promoter, was successfully transformed into the *Lb. plantarum* PA21 without any signs of incompatibility. Successful expression of GFP as a reporter was evaluated by Western blotting. The stability of pMG36e-gfp in *Lb. plantarum* PA21, without antibiotic selection, was monitored over 100 generations of growth in MRS broth medium to ensure the ability of the cells to harbor the plasmid. No loss of the plasmid was observed over this period, indicating very high stability. With this, it was shown that PA21 could potentially be used as a genetic modification tool, and represents an ideal candidate to design novel strategies for biological control of various pathogens specifically in the biofilm mode of growth.

## Conclusion

These results support the conclusions that *Lb. plantarum* PA21, a very potent biofilm producer provided specific local micro-environments which were favorable to some pathogen or spoilage microorganisms. Expression of GFP as a reporter allowed us to identify the strain with the potential to express heterologous proteins of interest. The results presented here can be used to support the studies aimed at developing new protective cultures with novel, existing or new combinations of genes, whose specific properties would devise ways in eliminating undesirable biofilm.

## Methods

### Isolation and identification of lactic acid bacteria

The *Lactobacillus* used in this study was isolated using standard microbiological procedures from the tropical plant *P. amaryllifolius*, which is commonly used in Southeast Asian cuisines [47]. The fermentation of 49 sugars and poly-alcohols (control) was carried out using the API 50 CHL kit (BioMérieux, Montalieu—Vercieu, France) incubated at 30°C. A rapid microtiter plate adherence test [48] was used to identify biofilm forming lactobacilli. Each well contained 2 ml of MRS broth (24-well microtiter plate; Nunc, Denmark) with 2% (v/v) inoculum of an overnight isolated culture that was incubated aerobically at 30°C for 1–2 days. After incubation, 500 µl of 0.21% (w/v) crystal violet staining solution (Fisher scientific Inc. USA) was added to each well and incubated at room temperature for 10 min. Microtiter plate wells were rinsed with 2 ml distilled water to remove unattached cells and residual dye.

### Determination of organic acid concentration

Three independent cultures of locally isolated *Lactobacillus* were used to determine the lactate and acetate concentration. For each sample, 1 ml of culture was centrifuged to harvest the cells, and the supernatant was collected. The glucose and lactate concentrations were measured with a Pico TRACE glucose-lactate analyzer (Trace analytics, Germany) according to the manufacturer’s protocol. The acetate concentration was determined using an Acetic Acid kit (Boehringer Mannheim/R-biopharm). The manufacturer-supplied standards were used as controls.

### Bacterial strains, plasmids and standard genetic manipulation techniques

The reference *Lb. plantarum* subsp*. plantarum* ATCC 14917 [49] and new isolate were grown in MRS agar and MRS broth (pH = 6.5 ± 0.2) (Merck, Germany) at 35°C without shaking under aerobic conditions for 1–3 days. Pure cultures of *Salmonella enterica* (Institute for Medical Research, Malaysia)*, Bacillus cereus* (Institute for Medical Research, Malaysia)*, Pseudomonas fluorescens* ATCC 13525 and *Aeromonas hydrophila* ATCC 7965 were chosen as representative Gram-positive and Gram-negative food-borne pathogenic and spoilage bacteria. *S. enterica*, *P. fluorescens* and *A. hydrophila* were cultured in tryptic soy agar or broth (pH: 7.3 ± 0.2) (Merck, Germany) and *B. cereus* in nutrient agar or broth (pH: 7.0) (Merck, Germany). All strains were maintained as stock cultures at −80°C in the respective cultivation broth containing 20% (v/v) glycerol (Merck, Germany).

All cloning steps were conducted according to standard procedures as described previously [50]. *Escherichia coli* TOP10 (Invitrogen) was grown at 37°C in Luria–Bertani (LB) broth with vigorous shaking at 200 rpm. PCR reactions were performed in a PCR Master Cycler (Eppendorf, Germany) with *Pfu* DNA polymerase (Promega Corp., Madison, WI, USA), as recommended by the polymerase supplier. *E. coli*-*Lactobacillus* shuttle vector pMG36e (generously gifted by Prof. Dr. Kees Leenhouts) and pGEM-T easy vector (Promega Co., USA) were used for protein expression and the cloning of PCR products, respectively. Chemically competent *E. coli* TOP 10 was transformed using the protocol provided by the supplier. Lactobacilli were transformed according to the protocol of Teresa et al. [51]. Ampicillin and erythromycin were added to final concentrations of 150, and 5 μg/ml, respectively. *E. coli* recombinants were screened by the addition of 0.004% (w/v) of 5-bromo-4-chloro-3-indolylb-d-galactopyranoside (X-gal), while the *Lb. plantarum* transformants were screened based on the erythromycin resistance selection marker. Plasmid DNA from *E. coli* and lactobacilli was isolated using a High Yield Plasmid Mini kit (Yeastern Biotech Co., Taiwan). DNA was extracted and purified from agarose gels using the Wizard SV Gel and PCR Clean-Up System kit (Promega Co., USA). The total genomic DNA was extracted and purified with Master Pure Gram Positive DNA purification kit (Epicentre Biotechnologies., USA). All PCR-derived DNA fragments were sequenced using the ABI 3730XL DNA analyzer (Bioneer Co., Korea).

### Phylogenetic analysis

The16S rDNA gene fragment was amplified from total genomic DNA using conserved primers 16sF-GCG GCG TGC CTA ATA CAT GC and 16sR -ATC TAC GCA TTT CAC CGC TAC close to the 3′ and 5′ ends [52].The PCR products of the 16s rDNA were purified and ligated into pGEM-T easy vector and then transformed to TOP10 chemically competent *E. coli*. All cloning steps and plasmid DNA isolation were conducted according to standard procedures as described previously.

The pGEM-T vector containing 16s rDNA regions of the LAB were extracted and sequenced using the ABI 3730XL DNA analyzer (Bioneer Co., Korea). Sequence similarity and database searches of DNA sequences or DNA-derived protein sequences were carried out using the BLASTN, BLASTP and BLASTX programs at the National Center for Biotechnology Information (http://www.ncbi.nlm.nih.gov/) [53]. The relationship between the bacterial strains was further analyzed with a phylogenetic tree using the MEGA 4.1. Program [54]. For phylogenetic analysis, multiple alignments of protein or nucleotide sequences were constructed using the program MAFFT 6.0 [55] and edited using BioEdit [56]. Trees were constructed based on the neighbor-joining method.

### Antibiotic susceptibility test

Pure culture colonies of lactobacilli were inoculated in MRS broth at 35°C for 24 h. The *Lactobacillus* strains were subcultured on MRS agar plates with sterile cotton swabs and allowed to air-dry. The susceptibility pattern to 10 antibiotics (Table 1) was assessed using the agar-disc diffusion method with minor modification including the relevant quality control strain [57]. The antibiotic discs (Oxoid) were placed on the agar, and the cultures were incubated at 37°C overnight. The diameter of the inhibition zone surrounding the antibiotic discs was measured. The test was carried out twice independently, and the average of the inhibition zone diameters was calculated. A plate containing only MRS was spread in tandem with the same overnight culture for a controlled comparison.

### Biofilm growth study

The assay to grow and quantitate *Lactobacillus* biofilm was prepared in 96-well microtiter plates (Nunc 96-well polystyrene microtiter plates, Denmark) under various environmental conditions, including variations in temperature and incubation time with the method of O’Toole et al. [58], which has been used for several other bacterial species. Scanning Electron Microscopy (SEM) was employed to capture biofilm cells grown on glass coverslips (12 mm diameter; Electron Microscopy Science, Hatfield, PA, USA) according to the method of Sturme et al. [59] with some modifications. Biofilms formed on glass coverslips were rinsed and fixed in 4% (w/v) glutaraldehyde for 12–24 h at 4°C. The fixed bacteria were rinsed three times for 10 min in 0.1 M sodium cacodylate buffer, then post fixed in 1% (w/v) osmium tetroxide at 4 °C. The coverslips were washed again with 0.1 M sodium cacodylate buffer for 3× of 10 min each and then dehydrated using acetone solutions of 35% (v/v), 50% (v/v), 75% (v/v), and 95% (v/v) for 10 min each and 100% (v/v) for three 15-min periods. To observe planktonic cells, bacterial cells grown in suspensions were subjected to same methods as those in the biofilm before being transferred to an Isopore 0.2-μm membrane filter (Millipore, USA). After critical point drying, the biofilms and dehydrated cells were sputter-coated with gold. Images were taken with an S4300SE/N scanning electron microscope (Philips XL30 ESEM, Institute Bioscience, UPM).

### Antimicrobial activity of LAB biofilms

The antibacterial effects of the LAB biofilm on the early development of food spoilage and pathogens were investigated according to the method of Guerrieri et al. [11]. The cells were grown in MRS broth and centrifuged at 2,900×*g* for 20 min at 4°C. The supernatant was removed, and the pellets were re-suspended in 5 ml of fresh MRS broth. After three washes, the final suspensions were diluted to a concentration of approximately 10^6^ CFU/ml. Biofilms were grown using 12-well microtiter plates. Two milliliters of *Lb. plantarum* PA21 suspensions in MRS broth were inoculated in each well and incubated for 7 days at 30°C to allow the adhesion and formation of mature biofilm in the well. Fifty percent of the growth medium was replaced with fresh broth every 48 h. After 7 days, the suspensions were removed, and the wells were washed three times with 1 ml of sterile saline solution (NaCl 0.85% w/v). A total of 2 ml of overnight cultures of *S. enterica, B. cereus*, *P. fluorescens* and *A. hydrophila* in their respective growth media were added to yield a final bacterial count of approximately 10^6^ CFU/ml and incubation continued for 6 days. For the planktonic bacterial enumeration, serial tenfold dilutions were spread on MRS agar plates for specific growth of PA21, on triptic soy agar plates for specific growth of *S. enterica,P. fluorescens* and *A. hydrophila* and nutrient agar plates for specific growth of *B. cereus* under the appropriate culture conditions [11, 60]. At the same time, the pH of suspension was determined using a Sartorius pH meter (Sartorius Ltd, Germany). Three wells incubated with each pathogen were washed three times before the biofilm was scraped off to evaluate the viable counts of microorganisms adhered to the biofilm. Serial tenfold dilutions were spread on agar plates and incubated using the same procedure. An additional control consisting of mixed cultures of PA21 and pathogens in the absence of *Lactobacillus* biofilm was also conducted to gain further insight into the importance of PA21 biofilm. Cell viabilities were assessed in three independent biological experiments.

### Generation of GFP construct

To construct pMG36e-GFP, a 717 bp DNA fragment encoding for GFP protein was amplified by PCR amplification using the primers FGFP (AGAGCTCCGATGAGTAAAGGCGA) and RGFP (CCAAGCTTTTATTTGTAT-AGTTCATCC), which correspond to the *gfp* sequence from plasmid BL21 (DE3) pLysS pet 32b(+) GFP (obtained from Microbial Biotech Laboratory, UPM). The primers included *SacI* and *HindIII* restriction sites on the ends to facilitate cloning. The amplified fragments were then cloned into pMG36e following digestion with the same enzymes to construct the expression vector pMG36e-GFP. The ligation mixture was transformed in *Lb. plantarum* PA21 competent cells, and Erm-resistant colonies were subjected to colony PCR with oligonucleotides that flank the pMG36e multiple cloning site. Insertion was verified by restriction digest analysis, and the integrity of the sequence was confirmed by sequence analysis.

### Plasmid stability

To test the stability of a plasmid under non-selective conditions, an overnight culture of *Lb.plantarum* PA21 harboring pMG36e-gfp was diluted (1:100) in MRS broth. The growth phases for *Lb. plantarum* PA21 carrying pMG36e-gfp were confirmed by calibrating the OD600 nm readings against CFU counts and the doubling time was calculated. The stability of the plasmid was tested based on a previously described method [61] with some modifications. Based on the number of generations in 24 h, the cells were maintained in the exponential phase for more than 100 generations. A volume of 100 μl of overnight culture of *Lb. plantarum* PA21 harboring pMG36e-gfp was inoculated into 100 ml MRS broth in the absence of antibiotic until 100 generations were achieved. Plasmid survival was assessed by comparing duplicate colony counts at the end of each 20-generations period on selective and non-selective plates. The percentage of plasmid stability was determined as the percentage of Erm-resistant colonies relative to the total number of viable colonies.

### Western blot analysis

An overnight culture of *Lb. plantarum* PA21 harboring pMG36e-gfp was diluted (1:40) in MRS medium supplemented with erythromycin, grown to an early-exponential phase (OD_600=_ 0.7) and harvested for protein analysis. Soluble protein extracts were collected following a procedure by Koistien et al. [57]. The proteins were quantified using the Bradford method. Western blot analysis was performed with a SDS-PAGE electrophoresis system using primary antibody *(*Anti-GFP Rabbit pAb; Calbiochem) diluted 1:2,000 in 0.01% (v/v) Tris-Buffered Saline Tween-20 (TBST) [61]. Nitrocellulose was then washed 4 times in 0.01% (v/v) TBST, and incubated with secondary antibody (Goat Anti-Rabbit IgG Alkaline phosphatase; Calbiochem) at a dilution rate of 1:5,000 in 0.01% (v/v) TBST for 2 h, washed again and developed.

### Statistical analysis

The investigations in this study were conducted as factorial experiments based on CRD (Completely randomized design). Three replicates were prepared for each biofilm and planktonic sample. The means were compared using Duncan’s Multiple Range Test (DMRT). The statistical analysis was performed using the SAS 9.2 software. All tests were carried out at confidence level 0.01.

## References

[1] Flemming H-C, Wingender J (2010). The biofilm matrix. Nat Rev Microbiol.

[2] Simoes M, Bennett RN, Rosa EAS (2009). Understanding antimicrobial activities of phytochemicals against multidrug resistant bacteria and biofilms. Nat Prod Rep.

[3] Vieira MJ, Melo LF, Pinheiro MM (1993). Biofilm formation: hydrodynamic effects on internal diffusion and structure. Biofouling.

[4] Bremer PJ, Fillery S, McQuillan AJ (2006). Laboratory scale Clean-In-Place (CIP) studies on the effectiveness of different caustic and acid wash steps on the removal of dairy biofilms. Int J Food Microbiol.

[5] Gram L, Bagge-Ravn D, Ng YY, Gymoese P, Vogel BF (2007). Influence of food soiling matrix on cleaning and disinfection efficiency on surface attached *Listeria monocytogenes*. Food Control.

[6] Dogan B, Boor KJ (2003). Genetic diversity and spoilage potentials among *Pseudomonas spp*. isolated from fluid milk products and dairy processing plants. Appl Environ Microbiol.

[7] Sharma M, Anand SK (2002). Characterization of constitutive microflora of biofilms in dairy processing lines. Food Microbiol.

[8] Elhariry HM (2011). Biofilm Formation by *Aeromonas hydrophila* on green-leafy vegetables: cabbage and lettuce. Foodborne Pathog Dis.

[9] Lindsay D, Brozel VS, Mostert JF, Von Holy A (2002). Differential efficacy of a chlorine dioxide-containing sanitizer against single species and binary biofilms of a dairy-associated *Bacillus cereus* and a *Pseudomonas fluorescens* isolate. J Appl Microbiol.

[10] Kreske AC, Ryu J-H, Pettigrew CA, Beuchat LR (2006). Lethality of chlorine, chlorine dioxide, and a commercial produce sanitizer to *Bacillus cereus* and *Pseudomonas* in a liquid detergent, on stainless steel, and in biofilm. J Food Prot.

[11] Guerrieri E, de Niederhäusern S, Messi P, Sabia C, Iseppi R, Anacarso I (2009). Use of lactic acid bacteria (LAB) biofilms for the control of *Listeria monocytogenes* in a small-scale model. Food Control.

[12] Speranza B, Sinigaglia M, Corbo MR (2009). Non starter lactic acid bacteria biofilms: a means to control the growth of *Listeria monocytogenes i*n soft cheese. Food Control.

[13] Levri KM, Ketvertis K, Deramo M, Merenstein JH, D Amico F (2005). Do probiotics reduce adult lactose intolerance? A systematic review. J Fam Pract.

[14] Reid G, Anand S, Bingham MO, Mbugua G, Wadstrom T, Fuller R (2005). Probiotics for the developing world. J Clin Gastroenterol.

[15] Bongaerts GPA, Severijnen R (2005). Preventive and curative effects of probiotics in atopic patients. Med Hypotheses.

[16] Viljanen M, Savilahti E, Haahtela T, Juntunenen-Backman K, Korpela R, Poussa T (2005). Probiotics in the treatment of atopic eczema/dermatitis syndrome in infants: a double-blind placebo-controlled trial. Allergy.

[17] Amdekar S, Dwivedi D, Roy P, Kushwah S, Singh V (2010). Probiotics: multifarious oral vaccine against infectious traumas. FEMS Immunol Med Microbiol.

[18] Delcenserie V, Martel D, Lamoureux M, Amiot J, Boutin Y, Roy D (2008). Immunomodulatory effects of probiotics in the intestinal tract. Curr Issues Mol Biol.

[19] Bernardeau M, Guguen M, Vernoux JP (2006). Beneficial lactobacilli in food and feed: long-term use, biodiversity and proposals for specific and realistic safety assessments. FEMS Microbiol Rev.

[20] Vandenbergh PA (1993). Lactic acid bacteria, their metabolic products and interference with microbial growth. FEMS Microbiol Rev.

[21] Corcoran B, Stanton C, Fitzgerald G, Ross R (2008). Life under stress: the probiotic stress response and how it may be manipulated. Curr Pharm Des.

[22] Gaggia F, Di Gioia D, Baffoni L, Biavati B (2011). The role of protective and probiotic cultures in food and feed and their impact in food safety. Trends Food Sci Technol.

[23] Cotter PD, Hill C (2003). Surviving the acid test: responses of gram-positive bacteria to low pH. Microbiol Mol Biol Rev.

[24] Giraffa G, Chanishvili N, Widyastuti Y (2010). Importance of *lactobacilli* in food and feed biotechnology. Res Microbiol.

[25] Simões M, Simões LC, Vieira MJ (2010). A review of current and emergent biofilm control strategies. LWT Food Sci Technol.

[26] An YH, Friedman RJ (2000) Handbook of bacterial adhesion: principles, methods, and applications. Springer Science & Business Media

[27] Neeser JR, Granato D, Rouvet M, Servin A, Teneberg S, Karlsson KA (2000). *Lactobacillus johnsonii* La1 shares carbohydrate-binding specificities with several enteropathogenic bacteria. Glycobiology.

[28] Coman MM, Verdenelli MC, Cecchini C, Silvi S, Orpianesi C, Boyko N (2014). In vitro evaluation of antimicrobial activity of *Lactobacillus rhamnosus* IMC 501, *Lactobacillus paracasei* IMC 502 and SYNBIO against pathogens. J Appl Microbiol.

[29] Watnick PI, Kolter R (1999). Steps in the development of a *Vibrio cholerae* El Tor biofilm. Mol Microbiol.

[30] Pan Y, Breidt F, Gorski L (2010). Synergistic effects of sodium chloride, glucose, and temperature on biofilm formation by *Listeria monocytogenes* serotype 1/2a and 4b strains. Appl Environ Microbiol.

[31] Bridier A, Sanchez-Vizuete P, Guilbaud M, Piard J-C, Naïtali M, Briandet R (2015) Biofilm-associated persistence of food-borne pathogens. Food Microbiol 45(Part B):167–178 10.1016/j.fm.2014.04.015 25500382

[32] Zhao T, Doyle MP, Zhao P (2004). Control of Listeria monocytogenes in a biofilm by competitive-exclusion microorganisms. Appl Environ Microbiol.

[33] Garcıa-Almendarez BE, Cann IKO, Martin SE, Guerrero-Legarreta I, Regalado C (2008). Effect of *Lactococcus lactis* UQ2 and its bacteriocin on *Listeria monocytogenes* biofilms. Food Control.

[34] Høiby N, Bjarnsholt T, Givskov M, Molin S, Ciofu O (2010). Antibiotic resistance of bacterial biofilms. Int J Antimicrob Agents.

[35] Mac Faddin JF (1999). *Biochemical tests for identification of medical bacteria*.

[36] Avelino Alvarez-Ordonez A, Begley M, Prieto M, Messens W, Lopez M, Bernardo A (2011). *Salmonella spp*. survival strategies within the host gastrointestinal tract. Microbiology.

[37] Sanjose C, Fernandez L, Palacios P (1987). Compositional changes in cold raw milk supporting growth of *Pseudomonas fluorescens* NCDO 2085 before production of extracellular proteinase. J Food Prot.

[38] Garde S, Gaya P, Medina M, Nunez M (2002). Autolytic behaviour of *Lactococcus lactis subsp. Cremoris* and *L. lactis subsp. lactis* wild isolates from ewes’ raw milk cheeses. Milchwissenschaft.

[39] Kives J, Guadarrama D, Orgaz B, Rivera-Sen A, Vazquez J, SanJose C (2005). Interactions in biofilms of *Lactococcus lactis* ssp. cremoris and *Pseudomonas fluorescens* cultured in cold UHT milk. J Dairy Sci.

[40] Mols M, Abee T (2008). Role of ureolytic activity in *Bacillus cereus* nitrogen metabolism and acid survival. Appl Environ Microbiol.

[41] Leslie AD (2011). Preventing biofilm formation using microbes and their enzymes. MMG 445 Basic Biotechnol eJournal.

[42] Rao MB, Tanksale AM, Ghatge MS, Deshpande VV (1998). Molecular and biotechnological aspects of microbial proteases. Microbiol Mol Biol Rev.

[43] Chassy BM, Flickinger JL (1987). Transformation of *Lactobacillus casei* by electroporation. FEMS Microbiol Lett.

[44] Römling U, Galperin MY, Gomelsky M (2013). Cyclic di-GMP: the first 25 years of a universal bacterial second messenger. Microbiol Mol Biol Rev.

[45] Kim Y, Oh S, Park S, Seo JB, Kim S-H (2008). *Lactobacillus acidophilus* reduces expression of enterohemorrhagic *Escherichia coli* O157: H7 virulence factors by inhibiting autoinducer-2-like activity. Food Control.

[46] McIntyre L, Hudson J, Billington C, Withers H (2007). Biocontrol of foodborne bacteria: past, present and future strategies. Food N Z.

[47] van den Berg DJ, Smits A, Pot B, Ledeboer AM, Kersters K, Verbake JM (1993). Isolation, screening and identification of lactic acid bacteria from traditional food fermentation processes and culture collections. Food Biotechnol.

[48] Reniero R, Cocconcelli P, Bottazzi V, Morelli L (1992). High frequency of conjugation in Lactobacillus mediated by an aggregation-promoting factor. J Gen Microbiol.

[49] Kubota H, Senda S, Nomura N, Tokuda H, Uchiyama H (2008). Biofilm formation by lactic acid bacteria and resistance to environmental stress. J Biosci Bioeng.

[50] Sambrook J, Russell DW (2001). Molecular cloning. A laboratory manual.

[51] Teresa Alegre M, Carmen Rodriguez M, Mesas JM (2004). Transformation of *Lactobacillus plantarum* by electroporation with in vitro modified plasmid DNA. FEMS Microbiol Lett.

[52] Weisburg WG, Barns SM, Pelletier DA, Lane DJ (1991). 16S ribosomal DNA amplification for phylogenetic study. J Bacteriol.

[53] Altschul SF, Gish W, Miller W, Myers EW, Lipman DJ (1990). Basic local alignment search tool. J Mol Biol.

[54] Tamura K, Dudley J, Nei M, Kumar S (2007). MEGA4: molecular evolutionary genetics analysis (MEGA) software version 4.0. Mol Biol Evol.

[55] Katoh K, Toh H (2008). Recent developments in the MAFFT multiple sequence alignment program. Brief Bioinform.

[56] Hall TA (1999) BioEdit: a user-friendly biological sequence alignment editor and analysis program for Windows 95/98/NT. In: Nucleic acids symposium series. Oxford Univeristy Press, pp 95–98

[57] Koistinen KM, Plumed-Ferrer C, Lehesranta SJ, Kärenlampi SO, Von Wright A (2007). Comparison of growth-phase-dependent cytosolic proteomes of two *Lactobacillus plantarum* strains used in food and feed fermentations. FEMS Microbiol Lett.

[58] O’Toole GA, Pratt LA, Watnick PI, Newman DK, Weaver VB, Kolter R (1999). Genetic approaches to study of biofilms. Methods Enzymol.

[59] Sturme MHJ, Nakayama J, Molenaar D, Murakami Y, Kunugi R, Fujii T (2005). An agr-like two-component regulatory system in *Lactobacillus plantarum* is involved in production of a novel cyclic peptide and regulation of adherence. J Bacteriol.

[60] Van der Veen S, Abee T (2011). Mixed species biofilms of *Listeria monocytogenes* and *Lactobacillus plantarum* show enhanced resistance to benzalkonium chloride and peracetic acid. Int J Food Microbiol.

[61] Noreen N, Hooi WY, Baradaran A, Rosfarizan M, Sieo CC, Rosli MI (2011). *Lactococcus lactis* M4, a potential host for the expression of heterologous proteins. Microb Cell Fact.

